# Human-centered digital ecosystem: safeguarding healthcare workers' sleep, ergonomics, and occupational health

**DOI:** 10.3389/fpubh.2026.1891645

**Published:** 2026-07-29

**Authors:** Donghui Ren, Yufang Qu, Hui Sun

**Affiliations:** Yantaishan Hospital, Yantai, Shandong, China

**Keywords:** artificial intelligence, cognitive shield, healthcare worker burnout, human-centered digital ecosystem, occupational health

## Abstract

Using Healthcare-Associated Infection (HCAI) surveillance as a representative model for high-burden administrative tasks, this Perspective argues that healthcare worker fatigue, sleep disruption, and poorly integrated digital systems threaten both occupational health and health-system resilience. Crucially, these problems should be addressed as failures of work-system design rather than deficits in individual resilience. We propose a Human-Centered Digital Ecosystem that places adequate staffing, circadian-aligned scheduling, protected recovery, and supportive organizational conditions before technological intervention. Within this ecosystem, artificial intelligence could potentially function as a task-specific “cognitive shield” by ranking, consolidating, or filtering low-value information before it reaches clinicians. However, AI's role should be strictly adjunctive; it is not a substitute for structural reforms such as increased hiring, fair compensation, and cultural shifts. AI-supported tools might be considered adjunctive and could be applied only for clearly defined and potentially avoidable administrative burdens after major organizational risks have been assessed and are being addressed in parallel. Their introduction should not delay, replace, or weaken investments in staffing, workload management, fair compensation, supportive leadership, and protected recovery. Implementation should proceed through staged validation, workforce co-design, continuous assessment of workload redistribution, and governance mechanisms protecting autonomy, equity, data security, and emotional privacy. We contend that digital interventions should be judged not only by technical accuracy or time saved, but also by their effects on after-hours work, cognitive workload, recovery, professional judgment, and the distribution of work across occupational groups. The purpose of digital transformation should be to remove avoidable work, not to compensate for preventable organizational deficiencies.

## 1 Introduction

Accelerated demographic transitions and the ongoing reconfiguration of healthcare infrastructures have engendered a critical occupational health crisis among medical

professionals. Clinicians endure chronic sleep restriction and circadian disruption, which compromise cognitive function and elevate cardiometabolic risk. Critically, this burnout epidemic signifies a structural deficit rather than inherent individual vulnerability. For instance, large-scale epidemiological data from the United States indicate a physician burnout prevalence of 45.8%; notably, possession of a medical degree constitutes an independent risk factor for burnout, underscoring the pathogenic nature of the clinical environment (1). Confirming this paradigm, a comprehensive meta-analysis of 52 studies has demonstrates that evidence-based structural and organizational interventions—such as workflow modifications and duty-hour limits—are significantly more effective at reducing overall burnout than individual resilience training alone (2).

Although AI has some promise in supporting clinical decision-making, burnout is a multifactorial phenomenon strongly influenced by inadequate staffing, excessive workload, insufficient financial compensation, and organizational culture. AI alone is unlikely to resolve these structural deficits. Healthcare systems therefore require an integrated framework that links occupational health, workflow redesign, digital support, and governance. Repetitive documentation, monitoring, and alert-processing tasks are treated here as representative sources of avoidable workload across clinical domains. Crucially, it must be unequivocally stated that algorithmic interventions cannot repair a broken organizational culture, compensate for chronic understaffing, or rectify inadequate compensation. AI might be strictly conceptualized as a ‘micro-dosed' digital intervention targeting specific cognitive burdens, and its deployment could be considered only after foundational structural hazards—such as circadian misalignment and unsafe nurse-to-patient ratios—have been substantially mitigated. The proposed Human-Centered Digital Ecosystem combines circadian-aligned work design with selectively deployed AI tools that reduce low-value information burden while preserving human oversight and equity. Furthermore, the manual execution of these diverse administrative and surveillance tasks is increasingly unsustainable due to cognitive overload, periodic system failures, and workflow disruptions. This may necessitate the development of a sustainable, human-centered digital ecosystem that could help automate standardized data acquisition and filtering low-value alerts across all clinical domains (3).

This Perspective conceptualizes AI-supported “cognitive shielding” as a task-specific approach in which digital systems rank, consolidate, or filter low-value information before it reaches clinicians. Unlike conventional decision-support tools that may add prompts to fragmented workflows, cognitive shielding is intended to operate subtractively by reducing non-actionable interruptions and prioritizing contextually relevant signals. Grounded in Cognitive Load Theory, this approach may help preserve limited working-memory capacity, but its benefits depend on validation, workflow integration, and human oversight (4). Because infrastructure constraints, biased data, and uneven model performance may distribute benefits and verification burdens unequally, implementation should also evaluate workforce burden, autonomy, privacy, safety, and health equity (5, 6).

### 1.1 The human-centered digital ecosystem: an occupational-health-oriented implementation framework

**Figure 1:**
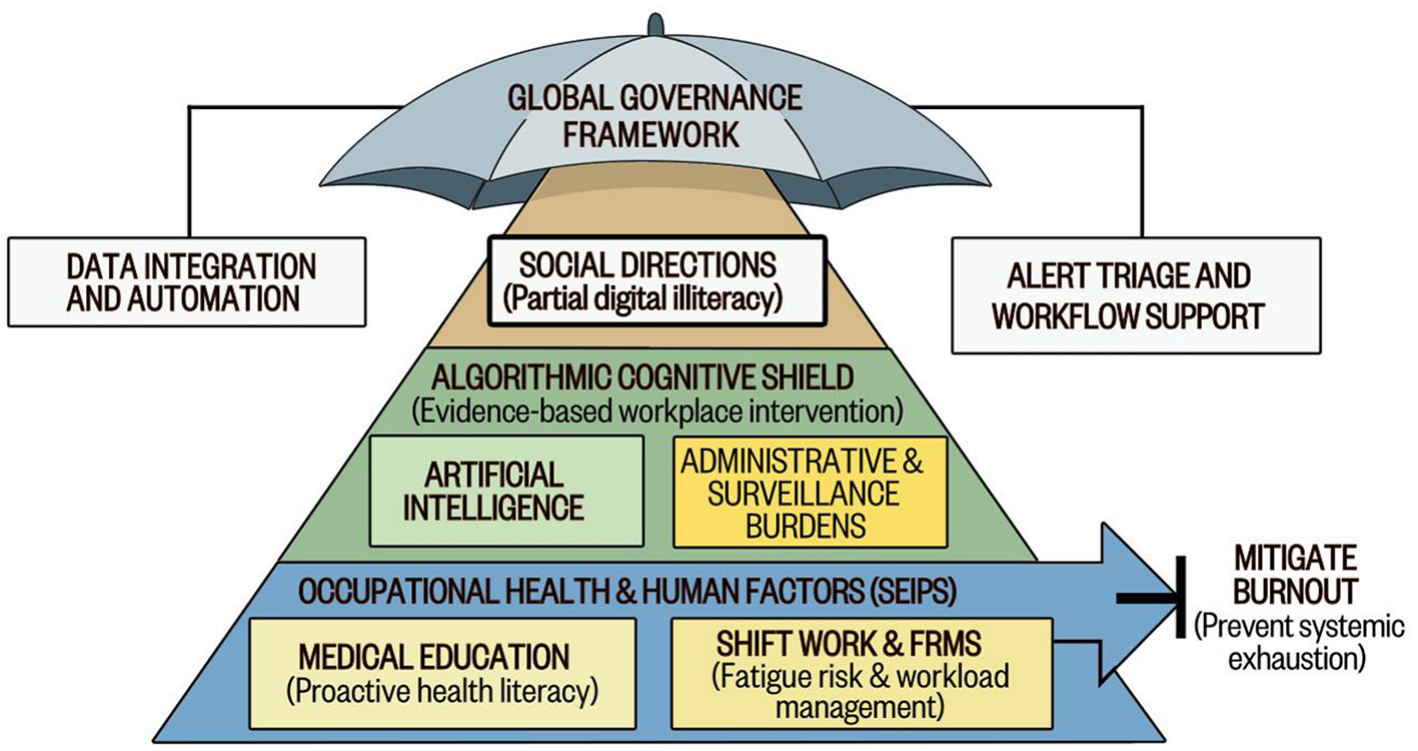
The human-centered digital ecosystem framework.

To examine the complex interplay among occupational exhaustion, work-system design, and digital transformation, this Perspective proposes the Human-Centered Digital Ecosystem as a conceptual and implementation framework (Figure 1). The framework is informed by evidence that poorly integrated digital technologies may increase rather than alleviate documentation demands, verification work, interruptions, and cognitive burden (3, 7, 8). It is not presented as a validated causal theory or as a replacement for established socio-technical models. Instead, it organizes structural, physiological, technological, and governance considerations into three interconnected domains intended to guide the design, implementation, and evaluation of digital interventions affecting the healthcare workforce.

The framework is grounded in the established systems engineering initiative for patient safety (SEIPS) model. SEIPS integrates Donabedian's structure–process–outcome perspective with human-factors engineering and emphasizes that occupational exhaustion, workflow disruption, and medical errors rarely originate from isolated individual failures. Rather, they emerge from interactions among core work-system components, including persons, tasks, tools and technologies, the physical environment, and organizational conditions (9). Subsequent developments, including SEIPS 2.0, have further emphasized work processes, adaptation, professional and patient journeys, and interactions across multiple work systems. Building on this system's perspective, the present framework examines how organizational conditions, physiological constraints, workflow design, and digital technologies may jointly influence both workforce wellbeing and patient safety.

While the SEIPS model provides a comprehensive, descriptive taxonomy of socio-technical variables affecting patient safety, the proposed Human-Centered Digital Ecosystem delivers a prescriptive, staged implementation logic. It specifically delineates the operational sequence required to protect clinicians' cognitive bandwidth and embed data solidarity before any algorithmic deployment. Rather than replacing or correcting SEIPS, the Human-Centered Digital Ecosystem framework builds on its work-system perspective by specifying an occupational-health-oriented sequence for digital intervention, extending from structural readiness and physiological protection to workflow simplification, task-specific digital support, and lifecycle governance. Its added contribution lies not in treating artificial intelligence as a fundamentally new work-system component, but in defining the conditions under which digital technologies may be introduced without intensifying occupational burden. In particular, the framework emphasizes that digital interventions should not substitute for adequate staffing, manageable workloads, supportive leadership, fair employment conditions, circadian-aligned scheduling, or protected recovery. These structural and physiological conditions should be assessed and addressed before or alongside technological implementation.

The framework also distinguishes between general digital transformation and narrowly defined AI-supported functions. Before introducing AI, healthcare organizations should first determine whether unnecessary work can be eliminated, whether workflows can be simplified, and whether interoperability or conventional automation can reduce duplicate data entry. AI might be considered only when a clearly defined residual burden remains and when its implementation could plausibly produce a net reduction in workload. Within this limited role, an AI-supported “cognitive shield” may rank, consolidate, or filter low-value information before it reaches clinicians, while preserving human oversight and final professional accountability.

Recent research on SEIPS 2.0 and human–AI teaming indicates that healthcare work increasingly spans multiple interacting systems and that the benefits of AI depend on task characteristics, interaction design, workflow integration, and user expertise (10–12). Human–AI collaboration should therefore not be assumed to produce cognitive complementarity automatically. Poorly designed systems may generate additional verification work, obscure responsibility, transfer burden to other occupational groups, or reduce situational awareness. The proposed framework responds to these concerns by linking digital intervention to explicit implementation conditions, including frontline co-design, staged validation, transparent escalation pathways, continuous monitoring of workload redistribution, and predefined criteria for modification or withdrawal.

The conceptual contribution of the Human-Centered Digital Ecosystem framework lies in connecting socio-technical work-system analysis with an explicit occupational-health implementation logic. The framework proposes that healthcare organizations should first evaluate structural readiness, then protect physiological limits and recovery, subsequently remove avoidable work, and only thereafter consider task-specific digital support for remaining burdens. Governance operates across all stages rather than being applied only after deployment. This approach complements SEIPS by translating a broad systems perspective into a staged framework focused specifically on the occupational-health consequences of digital transformation. Its practical contribution is the identification of workforce-centered evaluation criteria, including cognitive workload, after-hours electronic work, recovery opportunities, professional autonomy, task transfer, equity, and longer-term occupational-health outcomes.

To operationalize this staged logic, the proposed ecosystem (illustrated in Figure 1) is organized across three interconnected domains: (1) the Structural and Physiological Foundation, which addresses fundamental organizational conditions, fatigue risk management, and human recovery needs (1, 13–16); (2) the Workflow and Digital Support Layer, which focuses on eliminating high-burden tasks (e.g., manual HCAI surveillance) and deploying task-specific algorithmic assistance requiring prospective evaluation (6, 17–19); and (3) the Lifecycle Governance Layer, which ensures continuous oversight, data solidarity, and the protection of professional autonomy and emotional privacy (5, 20, 21). Rather than exhaustively detailing these components here, the subsequent sections of this Perspective will sequentially explore how each domain functions to safeguard the healthcare workforce. Ultimately, this framework does not position AI as the central remedy for healthcare worker burnout. Instead, it treats AI as one limited and conditional component of a broader socio-technical strategy whose primary aims are to remove avoidable work, protect recovery, preserve professional judgment, and prevent digital transformation from compensating for remediable organizational deficiencies.

## 2 Systemic failures in healthcare ergonomics

Although structural ergonomics and excessive cognitive loads are postulated as drivers of occupational health failures, the extant evidence base is constrained by a reliance on cross-sectional data, inherently precluding robust causal inference. Given that retrospective and observational studies harbor unmeasured confounding factors, these designs are generally inadequate for definitively establishing causation between specific ergonomic deficits and individual burnout (22). This critical finding challenges the prevailing bias that burnout stems from individual psychological fragility, pointing instead to systemic operational failures. One representative example of this broader administrative burden is manual HCAI surveillance, which may require infection-prevention personnel to repeatedly review microbiology results, clinical records, device data, admission information, and reporting fields. Although the magnitude of this burden varies across institutions and surveillance definitions, it illustrates how repetitive, compliance-driven monitoring can divert specialized personnel from direct prevention, feedback, education, and outbreak-response activities. Similar patterns occur in clinical documentation, quality reporting, medication reconciliation, and alarm management. These burdens may reduce professional efficacy and contribute to occupational exhaustion, although their independent causal effects remain difficult to establish (7, 8, 23, 24). Burnout should therefore be treated as a multifactorial organizational outcome rather than as a direct consequence of digital workload alone. Inadequate staffing, excessive workload, unstable schedules, insufficient compensation, limited decision latitude, unsupportive leadership, inequitable treatment, moral distress, and organizational cultures that normalize overwork may each contribute independently and interactively. While AI might mitigate the cognitive friction of digital interfaces, it remains incapable of addressing the root causes of moral distress, understaffing, and toxic leadership. Therefore, organizational investment in human capital must precede or parallel any algorithmic intervention (25).

### 2.1 Circadian misalignment and the “adaptation illusion”

Organizational pressures are compounded by circadian disruption and insufficient recovery. Repeated night work and unstable shift patterns can reduce sleep duration and impair alertness, cognitive performance, and physiological regulation, including severe endocrine and metabolic disruptions (26). Although healthcare workers may subjectively report adaptation across consecutive night shifts, objective measures such as the Psychomotor Vigilance Test often continue to show impaired performance (5, 13, 14, 16).

This discrepancy may be described as an “adaptation illusion”: subjective familiarity with night work does not necessarily indicate restoration of neurocognitive function, particularly during the biological night. From a patient-safety perspective, reliance on self-perceived alertness may therefore underestimate residual fatigue-related risk.

However, associations between circadian misalignment and adverse clinical outcomes should be interpreted cautiously. Much of the available evidence is derived from retrospective or observational cohorts, in which staffing levels, workload intensity, institutional culture, and other unmeasured factors may influence both fatigue exposure and clinical outcomes. These studies support concern but do not establish definitive causal relationships between circadian disruption and medical errors in real-world settings (22, 27). To fully contextualize the multifactorial nature of this occupational crisis, Table 1 summarizes the diverse structural stressors and their corresponding physiological degradation pathways across high-demand medical specialties.

**Table 1:** Occupational stressors, physiological erosion, and clinical outcomes across high-demand medical specialties.

Clinical setting/specialty	Core stressors & exposure patterns	Physiological & clinical outcomes (quantitative estimates)	Primary evidence source	Study design/level of evidence
Critical care (ICU) nursing	Chronic Desynchronization: 10-year fixed nocturnal duty; persistent biological-social misalignment.	Endocrine & Metabolic Failure: Significant circadian phase delay (avg. 5 h); >20% observed suppression of nocturnal melatonin; refractory hypertension associated with chronotherapeutic mismatch.	(15, 26, 29)	Systematic review & experimental study
Emergency medicine (ER)	Acute fragmentation: high-frequency rotation (≥16 night shifts/month); erratic sleep-wake cycles.	Neurocognitive Distress: associated with a 6.2-fold increase in self-reported medical error risk; 31% higher headache prevalence (aPR 1.31) correlated with night work independent of general stress; potential impaired prefrontal-parietal connectivity.	(13, 14, 31)	Prospective cohort & cross-sectional observational study
Pediatric & inpatient care	Immunological erosion: 5-year recurrent night shifts; chronic sleep debt (PSQI > 7).	Neuro-motor & biomechanical degradation: progressive decline in fine-motor kinematics; micro-expressions of fatigue identifiable via facial tracking. Immune risks: observed 37% reduction in NK cell activity; potential depletion of T-helper lymphocytes.	(15, 32)	Narrative literature review
Surgical specialties	Cumulative genomic strain: intrainstitutional heat stress; extreme cognitive load; shift accumulation.	Molecular & vascular damage: correlated with a 32% increased hypertension risk; peak neurological vulnerability (headache) observed on the 2nd consecutive night; markers of dna damage (instability of tp53/brca1 genes).	(13, 14, 32)	Prospective cohort & literature review

### 2.2 The digital “workload paradox” and human factors

Digital transformation is often introduced with the expectation that it may improve efficiency and reduce workload. In practice, poorly integrated systems may produce a “workload paradox” by replacing manual tasks with additional data entry, verification, troubleshooting, and compliance work. Automated hand-hygiene monitoring systems, for example, may increase the time required for compliance management when implementation is not aligned with existing workflows (3).

From a human-factors perspective, this problem arises when technology is introduced as an isolated solution without considering its effects on interconnected tasks, professional roles, and organizational processes. A system that reduces workload for one group may create new supervisory or corrective work for another. Similarly, transferring tasks from physicians to nurses or introducing additional digital monitoring may redistribute burden rather than reduce it (9).

Digital interventions should therefore be evaluated according to their net effects across the entire work system. Relevant outcomes include time spent on documentation, alert burden, verification work, after-hours electronic activity, cognitive workload, and task transfer between occupational groups. Excessive prompts and poorly calibrated alerts may impair attention and contribute to alert fatigue, particularly when systems increase recall or notification volume without improving meaningful clinical outcomes (28). Human-centered design should therefore prioritize subtraction of low-value work rather than the simple addition of new digital functions. In the absence of a rigorously human-centered design approach, conventional digital transformation merely displaces the workload onto digital interfaces without achieving genuine ergonomic relief.

### 2.3 AI governance and the proposed “cognitive shield”

AI deployment also raises occupational-health concerns when digital tools are used for employee surveillance rather than workforce support. Systems that infer mood, stress, engagement, or emotional state from facial expressions, voice, behavior, or passive sensor data may compromise professional autonomy and emotional privacy—the fundamental right to maintain the confidentiality of one's inner emotional state. The use of emotion-recognition or behavioral-monitoring technologies may also generate forced emotional labor if healthcare workers feel compelled to display algorithmically acceptable expressions or behaviors. Such systems may increase psychological strain, distrust, and perceived loss of control, particularly when monitoring purposes, data access, or consequences are unclear.

Governance should therefore distinguish between tools designed to reduce avoidable workload and systems designed to monitor employee behavior. AI-supported interventions should be subject to clear limitations on data collection, transparent purposes, meaningful worker participation, and safeguards against punitive or discriminatory use. Emotional privacy should be treated as an occupational-health consideration rather than solely as a data-protection issue (20, 25).

## 3 System optimization: redesigning for recovery

### 3.1 Upstream strategies: multidisciplinary system optimization

Multidisciplinary system optimization via upstream interventions constitutes the mandatory baseline for the proposed Human-Centered Digital Ecosystem. Instead of delegating the burden of extreme cognitive load to individual psychological resilience, healthcare institutions must deliberately design a “balanced work system” across the entire socio-technical spectrum (9). Operationalizing this balance requires establishing a formal Fatigue Risk Management System (FRMS) and addressing workload-staffing imbalances as evidence-based prerequisites prior to any digital intervention (29). Because adequate staffing and supportive leadership are profound drivers of clinical retention, failing to stabilize these elements before introducing digital tools risks exacerbating operational friction rather than providing ergonomic relief (30). While preventing fatigue-related harm justifies upstream investments from a health-economic perspective (31), organizations must also proactively manage workload through routine psychosocial risk assessments (8, 24) and Wellbeing Human Resource Management practices that foster psychologically safe environments (32). Robust quantitative evidence demonstrates that such structural adjustments yield significant reductions in overall physician burnout (2). Consequently, the implementation sequence must be strictly hierarchical: healthcare organizations must first stabilize staffing, scheduling, and cultural conditions; subsequently redesign workflows; and only thereafter consider AI for residual burdens. This ensures technology is not deployed to compensate for preventable organizational deficiencies.

### 3.2 Recovery-oriented work design and scheduling

Administrative workload reduction and circadian-aligned scheduling should be treated as complementary ergonomic interventions. Certain surveillance programs may require up to 18 h of administrative monitoring per week per 100 beds, illustrating how fragmented monitoring work can consume protected clinical time. Comparable burdens arise from documentation, quality reporting, compliance review, and alert management. At the same time, rapid shift rotations, such as “fast turns,” may provide fewer than 9.5 h of recovery and contribute to cumulative sleep debt. Together, excessive administrative workload and inadequate recovery time create mutually reinforcing pathways to occupational exhaustion (18, 33).

Correcting fragmented rest is merely the first step; extended, near-continuous periods of leave are equally essential for physiological recovery. Longitudinal evidence shows that approximately 60% of workers report improved health during vacations, with benefits persisting for at least 2 weeks after returning to work. To prevent chronic fatigue, schedules must incorporate protected, high-quality leave that encourages restorative activities (13, 15, 34, 35).

Beyond scheduling, ergonomic redesign must extend to the physical workplace environment, serving as another crucial defense against fatigue. For instance, while bright lighting during night shifts directly stimulates alertness and performance, it simultaneously suppresses melatonin and elevates cortisol, introducing severe long-term metabolic and carcinogenic risks. Advanced, evidence-based ergonomic interventions—such as implementing targeted lighting that filters out melatonin-suppressing wavelengths (specifically those below 480 nm)—can secure the safety benefits of enhanced vigilance without compromising the endocrine health of medical staff (29).

### 3.3 Task-specific AI support for residual burden

While scheduling interventions and Fatigue Risk Management Systems (FRMS) are supported by a comparatively established evidence base, the digital component of the proposed ecosystem remains more provisional. We use the term “AI-supported cognitive shielding” to describe a theoretical approach in which digital systems could rank, consolidate, or filter low-value information before it reaches the clinician. Unlike many conventional clinical decision-support systems (CDSS), which add alerts, prompts, or recommendations to existing workflows, this approach is intended to operate subtractively by reducing non-actionable interruptions and prioritizing contextually relevant signals. If appropriately designed, validated, and integrated into clinical workflows, such systems could reduce extraneous cognitive load, might help preserve working-memory capacity, and could support more focused clinical reasoning (4, 36).

However, these benefits should not be assumed. Inappropriately calibrated filtering could suppress clinically important information, create additional verification tasks, shift workload to other professional groups, or undermine clinicians' situational awareness and autonomy. The effects of AI-supported cognitive shielding on clinical reasoning, patient–clinician relationships, and healthcare worker wellbeing therefore require prospective empirical evaluation. Early real-world implementations of ranking-based alert management in high-volume public health monitoring provide preliminary proof of concept, but their findings should be interpreted as illustrative rather than as definitive evidence of improved occupational health outcomes.

One deployed public-health monitoring system provides early operational evidence for this subtractive, ranking-based approach. Joshi et al. (37) replaced conventional threshold-based alerting, which had generated large volumes of low-value notifications, with an unsupervised anomaly-detection system that ranked data points according to their deviation from contextualized expectations. In this human-in-the-loop implementation, the system was associated with a reported 54-fold increase in reviewer efficiency and improved user engagement by helping reviewers prioritize potentially high-impact events. These findings suggest that AI-supported ranking may help manage large volumes of signals more efficiently. However, because the system was implemented in public-health data monitoring rather than frontline clinical care, its findings cannot be assumed to generalize directly to bedside alarm management. The study also did not establish reductions in clinician burnout, sleep disruption, or longer-term occupational-health outcomes. Its relevance to the proposed cognitive-shield framework is therefore illustrative and hypothesis-generating rather than confirmatory (37). To translate this subtractive concept to bedside care, consider ICU alarm management. With 72%−99% of critical care alarms being false or non-actionable, alarm fatigue poses a severe risk to patient safety. An algorithmic “cognitive shield” could address this by utilizing machine learning to synthesize multi-parameter trends (e.g., heart rate, blood pressure) and filter sensor artifacts (38). By de-prioritizing clinically irrelevant signals, this system could help prevent sensory overload and might help preserve the exhausted clinician's working-memory for critical, human-in-the-loop interventions. To move beyond public health data and provide a direct clinical example, a real-world example of this subtractive approach could be found in recent implementations of algorithmic HCAI surveillance systems, which have been reported to replace manual electronic health record (EHR) screening. Some studies suggest that such interventions may have contributed to a reduction in the daily administrative hours of infection control practitioners, potentially decreasing their routine cognitive load. More broadly, human–AI teaming does not automatically produce cognitive complementarity. Emerging evidence suggests that performance may depend on the mode of integration, task characteristics, and the user's level of expertise. For example, simultaneous human–AI evaluation may perform differently from sequential review, and the benefits of AI support may vary between junior and senior clinicians. These findings indicate that an AI-supported cognitive shield should not be implemented as a uniform or static overlay. Instead, its interface, degree of automation, and escalation mechanisms may need to be calibrated to local workflows, task complexity, and user expertise. To reduce both inappropriate overreliance and premature rejection, the system could be designed to communicate uncertainty, offer accessible rationales, and allow clinicians to question, override, or escalate algorithmic outputs. Such design features may support active human oversight, although their effects on decision quality, cognitive workload, and professional autonomy require prospective evaluation (10, 12).

Current machine-learning systems remain brittle and often generalize poorly across clinical settings. Algorithms frequently exploit unintended confounders—such as associating the presence of a portable X-ray machine with pneumonia—rather than learning true clinical signals. Crucially, a recent systematic review revealed that only 6% of 516 eligible published studies evaluating diagnostic AI performed external validation. Consequently, continuous monitoring for “dataset shift” and site-specific retraining are mandatory to ensure these systems do not collapse when applied to novel clinical environments (15, 17, 18, 21, 28).

Furthermore, establishing the safety of healthcare software relies on the ability to inspect the program and understand its failure modes. Machine learning applications, however, often present a “black box” issue where underlying mechanisms remain invisible. This opacity creates a severe accountability gap; if frontline clinicians cannot comprehend the outputs generated by these algorithmic shields, they cannot appropriately justify their clinical actions and decisions (39).

### 3.4 Evaluation and continuous lifecycle governance

Before comprehensive clinical integration, algorithms must be deployed in a “shadow” or silent monitoring mode to identify hidden systemic errors and prevent patient harm prior to live deployment. Moving beyond isolated technical metrics, continuous performance monitoring must incorporate non-obtrusive, multidimensional evaluations of frontline cognitive burden and clinical utility to ensure the technology translates into meaningful ergonomic relief (6, 40, 41). Such holistic evaluations are essential to verify that algorithmic assistance successfully reduces extraneous load without creating new workflow disruptions (42).

Furthermore, evaluation must address the critical “work redistribution” phenomenon. Evidence from real-world implementations of ambient clinical intelligence demonstrates that while AI can reduce documentation burdens (e.g., “pajama time”), it may simultaneously introduce new supervisory tasks, such as verifying machine-generated accuracy (42, 43). Consequently, institutions must proactively ensure that time saved is genuinely converted into protected recovery or direct patient care, rather than being backfilled with additional administrative duties or patient appointments. Technology deployment must therefore be treated as an evidence-based workplace intervention, requiring continuous psychosocial risk assessments and active frontline co-design to protect clinical autonomy and job satisfaction (25).

Finally, translating this ecosystem into practice faces substantial implementation barriers, including siloed healthcare data, inconsistent semantic coding, and legacy IT infrastructures that impede true interoperability (28). Paradoxically, severe staffing constraints also hinder the adoption of labor-saving technologies, as overwhelmed clinicians lack the protected time required for digital literacy training. Combined with escalating cybersecurity risks associated with large-scale data aggregation, these challenges demand robust governance frameworks to protect patient privacy, data security, and professional autonomy (44). By rigorously navigating these barriers, healthcare systems could delegate low-value monitoring tasks to algorithmic systems. Ultimately, this could mitigate screen-induced fatigue and allow clinicians to refocus their attention on the humanistic aspects of care, potentially enabling patients to be treated with empathetic attention rather than as mere data points (3, 19, 45).

## 4 Outlook and global governance

### 4.1 Interoperable digital infrastructures, workforce sustainability, and global equity

While local workflow redesign and AI-supported cognitive shielding may provide immediate ergonomic relief, the long-term sustainability of these interventions depends on interoperable digital infrastructures that reduce duplicate data entry and enable information to be reused across care delivery, quality improvement, workforce planning, and public health functions. National platforms such as Iran's INIS provide a domain-specific example of the value of standardized, cross-institutional data aggregation. By 2023, INIS had compiled data from more than 1,066 hospitals and 11 million admissions, demonstrating the scale at which interoperable systems can support epidemiological analysis and resource allocation (46). The transferable lesson, however, extends beyond infection surveillance: interoperable infrastructures could replace repetitive manual data extraction, potentially improve information quality, and may support coordinated decision-making across multiple clinical domains.

Accordingly, global digital-health governance should prioritize systems that minimize administrative duplication and digital fatigue rather than simply expanding the volume of data collected. Alongside the clinical and epidemiological outcomes generated through large-scale surveillance activities (23), implementation should be evaluated using workforce-centered indicators, including time spent on documentation, after-hours electronic work, alert burden, perceived autonomy, usability, and cognitive workload. Treating these measures as core performance outcomes would help ensure that digital integration produces genuine ergonomic relief rather than transferring hidden work to frontline staff.

However, unchecked algorithmic monitoring can perpetuate deeply embedded discrimination. Evidence suggests that AI-based workplace surveillance may disproportionately penalize healthcare workers in vulnerable groups, including women and minority staff. These workers may shoulder greater emotional strain as they navigate biased algorithmic judgments and the pressure of entrenched social stereotypes (20). This vulnerability is compounded by the capacity of artificial intelligence models to embed and reproduce historical and social biases at scale. A major driver of this problem is selection bias within the underlying training datasets; because machine-learning development requires large datasets, algorithms are frequently constructed using research databases that represent highly selected and relatively homogeneous populations. Consequently, when deployed in real-world settings, these systems may fail or produce lower levels of accuracy when applied to underrepresented demographic groups (38). Existing hospital mortality-prediction algorithms, for example, have demonstrated varying accuracy across ethnic groups, while dermatological AI models trained predominantly on fair-skinned populations have underperformed when evaluating skin lesions in patients with skin of color. Deploying such models without appropriate correction risks exacerbating existing healthcare inequalities (28). For healthcare workers, these performance disparities may also create unequal verification and supervisory burdens, particularly for clinicians serving underrepresented populations. Repeatedly identifying, correcting, and documenting unreliable algorithmic outputs can increase cognitive workload, moral distress, and distrust in digital systems, thereby transforming algorithmic bias into an occupational-health risk rather than solely a patient-equity concern (28, 38).

Conversely, when intentionally designed with health equity as a primary objective, AI could theoretically contribute to mitigating these structural biases rather than merely perpetuating them. By developing and comparing multiple models—with and without specific demographic factors such as age, race, ethnicity, gender, and religion—developers may identify and correct systematically biased clinical decisions. For instance, algorithmic shields could theoretically be engineered to reduce distortions embedded in historical data and potentially help equalize differential referral patterns among patients from different racial or ethnic groups who present with comparable symptoms (41). From an occupational-health perspective, such bias-mitigation mechanisms may also reduce the disproportionate checking, correction, and escalation work imposed on clinicians caring for underrepresented populations. Nevertheless, these benefits depend on continuous auditing and meaningful frontline participation, because poorly calibrated attempts at algorithmic de-biasing may introduce new forms of complexity and supervisory burden.

### 4.2 Value-based governance and data solidarity

To mitigate the risks at the intersection of technology and occupational health, global institutions must adopt a targeted, value-based governance framework. Digital integration should not revolve solely around data extraction business models; it must prioritize the public interest and protect the healthcare workforce. Digital tools that could offer tangible ergonomic relief without significant risks should be actively supported. Conversely, applications that provide marginal public benefit while posing significant risks to clinical safety and privacy must be strictly regulated (21). Beyond direct clinical safety, the deployment of artificial intelligence without adequate human mediation introduces profound cybersecurity vulnerabilities. Utilizing artificial agents for pervasive surveillance creates a new attack vector driven by “data diet” vulnerabilities, which can jeopardize fundamental civil rights. These cybersecurity weaknesses represent a severe threat to the proposed digital ecosystem because they are typically hidden and discovered only after damage has occurred, putting vital healthcare infrastructures, human security, and resource access at immense risk (38).

“Data solidarity” is advanced as a normative framework designed to equilibrate individual data rights with collective public benefit. While this model could offer a theoretical pathway for the equitable distribution of AI's benefits and risks, empirical examples of its successful implementation at scale remain limited. Formalizing such value-based governance mechanisms presents significant regulatory challenges that require further critical exploration (20, 21).

Within this value-based governance architecture, there exists a compelling imperative to codify “emotional privacy” as a fundamental occupational entitlement. As the healthcare system increasingly integrates emotional AI and passive sensing technologies, policy frameworks must prevent clinicians from facing pervasive behavioral monitoring and algorithmic emotional manipulation. Incorporating clauses related to emotional privacy into the data solidarity framework—and enforcing strict risk classifications under global regulatory standards—could proactively mitigate these profound sociotechnical hazards. Ultimately, this ensures strict adherence to the ethical boundaries of medical staff during digital integration (20).

### 4.3 Algorithm lifecycle and sociotechnical usability

To maintain continuous oversight of algorithms, global governance must integrate existing regulatory frameworks, such as the guidelines of the International Medical Device Regulators Forum and the structural requirements of the European Union's Artificial Intelligence Act (2024). Categorizing speculative AI-driven ‘cognitive shields' as Software as a Medical Device (SaMD) enables regulators to enforce stringent risk classifications and mandate continuous post-market surveillance. Without establishing such clear governance and regulatory frameworks, medical teams risk deploying outdated or biased algorithms, leading to unresolved legal liability and severe clinical safety issues. Without these usability optimizations, clinicians are highly likely to experience systemic dissatisfaction, leading to technological abandonment (21).

### 4.4 Reshaping occupational health and medical education

Sustainable healthcare development necessitates the optimization of occupational adaptation and structural job benefits. To effectively advance these systemic interventions, interdisciplinary collaboration must evolve into a shared, multi-level stakeholder framework. By engaging both internal actors (e.g., hybrid clinical line managers) and external entities (e.g., healthcare unions, professional associations, and government bodies), organizations can move beyond *ad-hoc*, reactive wellness initiatives (32). Early engagement of these stakeholders ensures that digital transformation directly addresses clinical fatigue and operational bottlenecks rather than merely shifting the burden. Consequently, global health governance must transition toward a dual-priority model of occupational safety. This approach mandates not only scientifically validated, evidence-based scheduling requirements but also the routine integration of sleep disorder education and screening into occupational health assessments. Such a systemic strategy is crucial for mitigating long-term cognitive impairment and cardiometabolic degradation, which remain critical challenges for the global healthcare workforce (16).

Within digital health governance, the adoption of personalized scheduling algorithms has been proposed as a potentially promising, albeit speculative, intervention. Interindividual physiological variability dictates that practitioners exhibit differential susceptibilities to shift work, with some experiencing severe neurological symptoms such as headaches. Utilizing intelligent systems to calibrate shift sequences based on personal health metrics could theoretically sustain the long-term occupational capacity of high-risk cohorts (14).

Furthermore, governance frameworks must extend upstream into medical education. Because the health risks associated with shift work peak during early career transitions, nursing and medical curricula must preemptively incorporate evidence-based sleep health education and digital health literacy. Academic leadership and clinical practitioners must collaborate to align clinical training with digital advancements. Cultivating cognitive coping strategies and physiological recovery skills prior to formal employment provides an essential, evidence-based foundation for long-term clinical sustainability and workforce retention (5, 15). Crucially, this preemptive training must evolve beyond basic digital literacy to encompass human-AI ‘meta-coordination.' Future curricula must train professionals to actively calibrate trust, manage algorithmic delegation, and navigate escalation protocols, ensuring that human clinicians consistently retain ethical authority and accountability in high-stakes clinical decision-making (12).

## 5 Conclusion

Healthcare worker burnout and occupational ill health arise from interacting structural, physiological, ergonomic, and cultural conditions. The Human-Centered Digital Ecosystem framework proposed in this Perspective should therefore be understood not as an AI-centered remedy, but as a staged implementation framework that places adequate staffing, manageable workload, fair employment conditions, circadian-aligned scheduling, protected recovery, and supportive leadership at its foundation. Within this broader system, AI-supported cognitive shielding might help reduce specific forms of avoidable documentation, monitoring, and alert burden, including those illustrated by HCAI surveillance.

The framework complements SEIPS by translating its work-system perspective into an occupational-health-oriented sequence for digital intervention. Its proposed contribution is to connect structural readiness, physiological protection, workflow simplification, task-specific digital support, and lifecycle governance within a single implementation logic. However, the framework itself has not yet been prospectively validated, and its AI-supported components remain largely conceptual. Current evidence is concentrated in narrow, context-specific applications and predominantly concerns short-term process or workload outcomes. Whether these interventions improve sleep, burnout, workforce retention, or longer-term occupational health therefore remains uncertain.

If implemented with meaningful frontline participation, transparent validation, privacy protection, equity safeguards, and predefined stopping rules, human-centered digital tools could become one component of a broader strategy to protect the healthcare workforce. Their role should be to remove avoidable work and support professional judgement, not to compensate for chronic understaffing, inadequate compensation, or harmful organizational cultures.

## Data Availability

The original contributions presented in the study are included in the article/Supplementary material, further inquiries can be directed to the corresponding author.
